# Putting the Pieces Together: Integrative Modeling Platform Software for Structure Determination of Macromolecular Assemblies

**DOI:** 10.1371/journal.pbio.1001244

**Published:** 2012-01-17

**Authors:** Daniel Russel, Keren Lasker, Ben Webb, Javier Velázquez-Muriel, Elina Tjioe, Dina Schneidman-Duhovny, Bret Peterson, Andrej Sali

**Affiliations:** 1 Department of Bioengineering and Therapeutic Sciences, Department of Pharmaceutical Chemistry, California Institute for Quantitative Biosciences (QB3), University of California, San Francisco, San Francisco, California, United States of America; 2 Raymond and Beverly Sackler Faculty of Exact Sciences, Blavatnik School of Computer Science, Tel Aviv University, Tel-Aviv, Israel; 3 Google, Mountain View, California, United States of America

## Abstract

A set of software tools for building and distributing models of macromolecular assemblies uses an integrative structure modeling approach, which casts the building of models as a computational optimization problem where information is encoded into a scoring function used to evaluate candidate models.

## Introduction

Building models of a biological system that are consistent with the myriad data available is one of the key challenges in biology. Modeling the structure and dynamics of macromolecular assemblies, for example, can give insights into how biological systems work, evolved, might be controlled, and even designed. Modeling can also suggest future experiments. Unfortunately, current publishing norms make it hard to build on published models, because such models are often not available in usable form and because it is hard to publish refinements of others' models. Here, we present steps towards a future in which a scientist can read a paper, download a script, add new data, and see how the new data improve the published model. Integrative structure modeling casts the building of structural models as a computational optimization problem, for which information about the assembly is encoded into a scoring function that evaluates candidate models. We describe our software suite, Integrated Modeling Platform, and invite members of the scientific community to use it, improve on it, and apply it to their own scientific problems of interest.

Numerous structures have to date been solved by using an integrative structural modeling approach. The structure of the 26S proteasome was determined from an electron microscopy (EM) map of the whole assembly, proteomics data about its subunit composition, and comparative protein structure models of the component proteins [1]. The structure of the bacterial type II pilus was assembled from sparse nuclear magnetic resonance (NMR) data and X-ray crystallographic structures of constituent proteins [2]. The structure of chromatin around the alpha-globin gene was assembled from so-called 5C data (chromosome conformation capture carbon copy) [3]. The value of integrative modeling is illustrated by its application to the yeast nuclear pore complex (NPC) [4],[5]. The sheer size and flexibility of the NPC makes it all but impossible to solve its molecular architecture by conventional atomic resolution techniques, such as X-ray crystallography. However, integrating information from multiple sources, including stoichiometry from protein quantification, protein proximities from subcomplex purification, protein positions from immuno-EM, sedimentation analysis that sheds light on protein and subcomplex shapes, and the overall NPC shape from EM, resulted in an ensemble of medium-resolution models. The models were summarized by a 3-D probability map, resembling an EM map and localizing the 456 constituent proteins with an average precision of approximately 5 nm. This map has revealed fundamental new insights into the function of the NPC as a gatekeeper controlling the entry into and exit from the nucleus of macromolecules, and also shed light on its evolution [4],[6]–[8].

**Figure 1:**
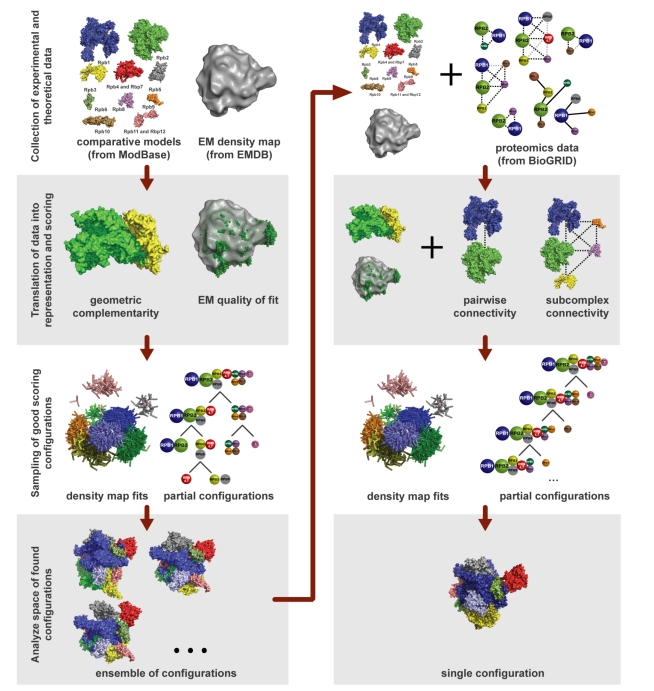
Integrative structure modeling of the human RNA Polymerase II [**10**]. The first round of modeling was performed using only the 2nm EM density map of the assembly from EMDB [51] and subunit comparative models from ModBase [47], on the basis of the crystallographic structures of the yeast RNAPII proteins. The data were found to be insufficient to uniquely resolve the structure. To overcome this challenge, protein interaction networks extracted from BioGrid [48] were added. The addition of these data resulted in a single structure. The scripts are available as part of IMP.

Integrative modeling entails a computational encoding of the standard scientific cycle of gathering data, proposing hypotheses, and then gathering more data to test and refine those hypotheses. It proceeds through repeated iterations of the stages of gathering information, choosing how to represent and evaluate models, finding models that score well, and analyzing the models and information (Figure 1; Box 1). The cycle terminates when a convergent ensemble of models is found fitting the current information and the models have been judged to be satisfactory [9]. When new information is gathered, whether by other scientists or other techniques, the cycle is resumed.

**Box 1. The Four Stages of the Integrative Modeling Cycle**

**Stage 1: Gathering Information**

Information is collected in the form of data from wet lab experiments, as well as statistical tendencies such as atomic statistical potentials, physical laws such as molecular mechanics force fields, and any other feature that can be converted into a score for use to assess features of a structural model.

**Stage 2: Choosing How To Represent And Evaluate Models**

The resolution of the representation depends on the quantity and resolution of the available information and should be commensurate with the resolution of the final models: different parts of a model may be represented at different resolutions, and one part of the model may be represented at several different resolutions simultaneously. The scoring function evaluates whether or not a given model is consistent with the input information, taking into account the uncertainty in the information.

**Stage 3: Finding Models That Score Well**

The search for models that score well is performed using any of a variety of sampling and optimization schemes (such as the Monte Carlo method). There may be many models that score well if the data are incomplete or none if the data are inconsistent due to errors or unconsidered states of the assembly.

**Stage 4: Analyzing Resulting Models and Information**

The ensemble of good-scoring models needs to be clustered and analyzed to ascertain their precision and accuracy, and to check for inconsistent information. Analysis can also suggest what are likely to be the most informative experiments to perform in the next iteration.

Integrative modeling iterates through these stages until a satisfactory model is built. Many iterations of the cycle may be required, given the need to gather more data as well as to resolve errors and inconsistent data.

The integrative approach has a number of advantages over informal or partial consideration of available information (Box 2). Fully realizing these advantages requires encoding modeling efforts into integrative modeling applications that consist of the scripts and the associated information. Adoption of integrative modeling can occur through a tight collaboration between a computational lab and an experimental lab, through direct adoption by an experimental lab, or by experimentalists modifying existing integrative modeling applications. To facilitate widespread adoption, we have developed the Integrative Modeling Platform (IMP) software package.

**Box 2. Advantages of the Integrative Structure Modeling Approach**

**Using New Information**

Integrative modeling makes it easy to take advantage of new information and new types of information, resulting in a low barrier for using incremental information that is generally not applied to structure characterization. Even when a single data type is relatively uninformative, multiple types can give a surprisingly complete picture of an assembly [9],[10].

**Maximizing Accuracy, Precision and Completeness**

Integrative models fit multiple types of information, and can thus be more accurate, precise, and complete than models based on the individual sources.

**Understanding and Assessing the Models**

By exhaustively sampling the space of models fitting the information, integrative modeling can find all models fitting the information, not only one. A full sampling of the models of a structure can improve the understanding of its function [49]. Because the data are encoded in scoring functions and the full set of models can be found, integrative modeling facilitates assessing the input information and output models in terms of precision and accuracy.

**Planning Experiments**

Integrative modeling provides feedback to guide future experiments, by computationally testing the impact of hypothetical datasets. As a result, experiments can be chosen to best improve our knowledge of the assembly.

**Understanding and Assessing Experimental Accuracy**

Data errors present a challenge for all methods of model building. Integrative modeling can detect inconsistent data as no models will exist that fit all the data. In addition, integrative modeling facilitates the application of more sophisticated methods for error estimation, such as Inferential Structure Determination [16].

**Box 3. Key Requirements for Integrative Modeling Infrastructure**

**Modular structure**

To allow a community of developers to easily add sources of information, sampling schemes or analysis methods, IMP is structured as a collection of self-contained modules that can be developed and distributed independently.

**Simple abstractions**

Having a set of simple common abstractions allows independently written modules to be used together. The restraint/scoring function abstraction provides one such, allowing arbitrary data to be combined. Representing models as collections of particles with associated data provides another, allowing easy mixing of coarse grained and atomic models.

**Easy sharing**

IMP provides a platform-independent high level scripting interface for writing integrative modeling applications from data to analysis; this reduces the burden of supporting the applications and so reduces the cost of sharing.

**Higher level entry points**

IMP provides a set of high level tools to facilitate application of established protocols to new systems. These include Multifit for assembling multiple subunits based on an EM density map, proteomics data and molecular docking [10], and FoXS for computing a SAXS profile of a given structure [50], both of which can be use via web interfaces, from Chimera [24] or from the command line.

## A Platform for Integrative Modeling

The IMP software package facilitates the writing of integrative modeling applications; the development of new model representations, scoring functions, sampling schemes, and analysis methods; and the distribution of integrative modeling applications.

In IMP, models are encoded as collections of particles, each representing a piece of the system. Depending on the data available, particles can be used to create atomic, coarse-grained, or hierarchical representations. It is straightforward to represent a protein at any resolution, from fully flexible atomic models (one particle per atom), to rigid bodies, to coarse-grained models consisting of only one or a few particles for the whole protein (see Figure 1 for a worked-through example, structural modeling of the human RNA polymerase II [10]). Different parts of the model can be represented differently, as dictated by the available information. Each particle has associated attributes, such as coordinates, radius, atom type, rigid body composition, residue information, and mass. If the attributes already in IMP are not sufficient, new attributes can be created and used similarly to the predefined ones. For example, for coarse-grained small angle X-ray scattering (SAXS) scoring, a scattering factor attribute could be associated with the particles representing amino acid residues.

Candidate models are evaluated by a scoring function composed of terms called restraints, each of which measures how well a model agrees with the information from which the restraint was derived. The restraints encode both what is known about structures in general and what is known about this particular structure. Thus, a candidate model that scores well is consistent with all used information. The precision and accuracy of the resulting model increases with the amount and quality of information that is encoded in the restraints. IMP's ever-growing set of scoring function types includes ones for SAXS profiles [11], proteomics data [9], EM images and density maps [10],[12], NMR spectroscopy [2], the CHARMM force-field [13], alignment with related structures [14], and a variety of statistical potentials [15]. IMP has been designed to make it easy for others to develop, use, and distribute new restraints. Other research groups are currently implementing restraints for various mass spectrometry measurements, SAXS, 5C data [3], and atomic structure prediction.

For experimental data, the scoring is generally implemented using a “forward model” [16], which simulates the measurements on the basis of the candidate model and then compares the simulated measurements to the actual measurements. For example, to evaluate the fit to an EM density map, a restraint uses the coordinates, radii, and masses of a set of particles representing the assembly to simulate its density map and then evaluates the cross-correlation with the experimental map.

As with most computational structure efforts, the main demand for computational time in integrative modeling comes from sampling models that satisfy the restraints (good-scoring models). IMP provides a wide variety of tools for building these sampling protocols, including optimization algorithms such as Monte Carlo [17] and conjugate gradients [18], the simplex optimizer from Gnu Scientific Library (GSL) [19], simulation schemes such as molecular dynamics and Brownian dynamics [20], and the Bullet rigid body dynamics engine (http://www.bulletphysics.com), as well as full sampling schemes such as the Gibbs sampler [16], replica exchange [21], and a divide-and-conquer sampler called DOMINO [22].

Finally, IMP provides a variety of tools for comparing, clustering, and analyzing models. These tools can be used to check for quality-of-fit, the existence of multiple states of the system [3], and inconsistent information. Models can be clustered on the basis of root-mean-square deviation (RMSD), placement score [11], and various other metrics. Supported clustering algorithms include k-means, centrality betweenness clustering [23], and simple binning. The resulting clusters and the constituent models as well as restraints can be exported to Chimera [24] and Pymol [25] for visual inspection and further analysis.

IMP has been used to produce a number of models; for example, a eukaryotic ribosome [26], a mammalian ribosome [27], a ryanodine receptor channel [28], the 26S proteasome [1], the Hsp90 chaperonin [29], the TRiC/CCT chaperonin [30], the actin-scruin complex [31], chromatin [3], and the NPC [4]. More information about IMP can be found at http://integrativemodeling.org/. The website provides a technical introduction, a tutorial, as well as a variety of examples to help users get started. In addition, it contains nightly tests, user and developer email lists, a wiki, and a bug tracker.

## Towards Open Structure Modeling

Publication of macromolecular structures has evolved from printed words and pictures to include deposition of coordinates in the Protein Data Bank [32], and more recently deposition of raw input data such as X-ray scattering factors [32], NMR restraints [33], and EM particle images [34]. However, the conversion of the raw data to the final structures is often only briefly described and all too rarely available in a directly usable form [35]–[37], making reproduction and use of the published results laborious or even impossible.

If published papers included integrative modeling applications, a wide variety of researchers would benefit. In particular, experimental labs, which are unlikely otherwise to go through the effort of modeling systems themselves, would be able to use the state-of-the-art model to plan experiments by simulating potential benefits gained from new data. It would also be easy to see how much each new measurement contributes to and fits with the current model. Other computational groups could more easily experiment with new scoring, sampling, and analysis methods, without having to reimplement the existing methods from scratch. The common abstraction would make it easier to mix and match parts of other modeling packages [13],[14],[16],[38]–[46] to improve the applications of integrative modeling. Finally, the authors themselves would maximize the impact of their work, increasing the odds that their results are incorporated into future modeling.

## Abbreviations

EM: electron microscopy
IPM: Integrative Modeling Platform
NPC: nuclear pore complex
SAXS: small angle X-ray scattering
